# Antimicrobial Stewardship Programs in Resource Constrained Environments: Understanding and Addressing the Need of the Systems

**DOI:** 10.3389/fpubh.2020.00140

**Published:** 2020-04-28

**Authors:** Ashish Kumar Kakkar, Nusrat Shafiq, Gurpreet Singh, Pallab Ray, Vikas Gautam, Ritesh Agarwal, Jayashree Muralidharan, Pankaj Arora

**Affiliations:** ^1^Department of Pharmacology, Post Graduate Institute of Medical Education and Research, Chandigarh, India; ^2^Department of General Surgery, Post Graduate Institute of Medical Education and Research, Chandigarh, India; ^3^Department of Medical Microbiology, Post Graduate Institute of Medical Education and Research (PGIMER), Chandigarh, India; ^4^Department of Pulmonary Medicine, Post Graduate Institute of Medical Education and Research (PGIMER), Chandigarh, India; ^5^Advanced Pediatrics Centre, Post Graduate Institute of Medical Education and Research (PGIMER), Chandigarh, India; ^6^Department of Hospital Administration, Post Graduate Institute of Medical Education and Research (PGIMER), Chandigarh, India

**Keywords:** antimicrobial resistance (AMR), antimicrobial stewardship (AMS), antimicrobial therapy, guidelines & recommendations, prospective audit and feedback, LMIC = low- and middle-income countries

## Abstract

World Health Organization (WHO) has identified antimicrobial resistance as one of the top 10 threats to public health. The agency has formulated a global action plan to tackle antimicrobial resistance by reducing incidence of infectious diseases, increasing knowledge and awareness and promoting rational use of antimicrobials amongst other measures. While the core elements of successful antimicrobial stewardship (AMS) programs are much publicized, there application in resource limited settings is fraught with several challenges. The key limiting factors include lack of clear political commitment, inadequate funding, overcrowded healthcare systems, lax legal and regulatory frameworks, non-uniform access to diagnostics, absence of electronic health record systems, limited knowledge and awareness especially with existence of multiple systems of medicines, issues with access to quality assured medicines, in-house pharmacies, and shortage of trained manpower. Since these implementation-impeding issues may differ considerably from those experienced in developed economies, intervention efforts in low- and middle-income countries (LMICs) need to address the context and focus on the root causes prevailing locally. In this article, we review the evidence highlighting the magnitude of these challenges and suggest feasible models with effective application. We also share the evidence from our center where we have contextualized the core elements to resource constrained settings. These domains include delivering prospective audit and feedback, prescriber education, development of evidence-based and implementable guidelines, and optimization of surgical antibiotic prophylaxis. However, there is a tremendous need for scaling up, extending outreach and honing these models while at the same time, addressing the existing strategic challenges that curtail the full potential of global antimicrobial stewardship.

## Introduction

Antimicrobial resistance is a growing public health challenge worldwide that has been identified as one of the top 10 threats to global health by World Health Organization (WHO) in 2019 (1). A 2016 report on antimicrobial resistance (AMR) estimated that by 2050, nearly 10 million deaths per year and a overall GDP loss of 100 trillion could be attributable to drug resistant infections if appropriate measures are not instituted (2). Furthermore, the greatest—direct as well as indirect impact of AMR will be felt by Low- and Middle-Income Countries (LMICs). Even though the development of antimicrobial resistance is considered to be a biological evolutionary response to antibiotic exposure, the situation is worsened by interplay of several drivers including human and animal misuse and overuse, environmental contamination, healthcare transmissions, and suboptimal diagnostics, vaccinations and pharmaceutical quality (3, 4). According to a recent analysis, between 2000 and 2015, global consumption of antibiotics increased 65% from 21.1 to 34.8 billion defined daily doses (DDDs), while the antibiotic consumption rate increased by 39% from 11.3 to 15.7 DDDs per 1,000 individuals per day over the same period. The increase in global consumption was primarily driven by increased utilization in LMICs with India, China and Pakistan being the leading antibiotic consumers among them. Four of the top six countries with the highest antibiotic consumption rates were LMICs including Turkey, Tunisia, Algeria and Romania. According to these estimates the antimicrobial consumption in LMICs is rapidly converging to the rates prevalent in high income countries. If this trend goes unabated, global antibiotic consumption in 2030 is poised to be up to 200% greater than the 42 billion DDDs estimated for 2015 (5).

The World Health Organization in 2015 formulated a global action plan to tackle antimicrobial resistance by reducing incidence of infectious diseases, increasing knowledge and awareness of AMR and promoting rational use of antimicrobials amongst other measures (6). Under the global action plan, the agency has recognized antimicrobial stewardship (AMS) programs as one of the key interventions especially in the wake of dwindling new drug pipeline as far as anti-infective agents are concerned. Antibiotic stewardship as a strategy, can be viewed as a coordinated set of actions aimed at promoting prudent use of antimicrobials, with the ultimate goal of optimizing clinical outcomes while minimizing the unfavorable consequences including resistance selection as well as adverse drug reactions (7, 8). A recently updated Cochrane review based on more than 200 studies from diverse settings, found that antimicrobial stewardship interventions in hospitals result in greater compliance with treatment guidelines, reduced total duration of antimicrobial treatment, and lead to shorter length of hospital stays without adversely impacting patient mortality (9). Another systematic review and meta-analysis by Schuts et al. supported application of several AMS interventions including guideline directed use of empiric antimicrobials, de-escalation, switching from intravenous to oral therapy, antibiotic restrictions, therapeutic monitoring and bedside consultations in terms of improved patient outcomes, reduced costs as well as occurrence of adverse events (10). Despite the evident advantages and gains, managing successful antimicrobial stewardship programs in healthcare institutions is challenging in general and even more so in resource constrained environments. The present article attempts to highlight the key limitations faced by the relatively nascent AMS programs in LMICs that prevent realization of full potential of these imperative strategies. We also propose ways to address these problems and suggest solutions based on available literature and drawn from own experience of implementing an AMS program in one of the largest public funded tertiary level healthcare facility in North India and a leading medical research institute of the country.

## AMS Challenges in Resource Limited Settings

In general, healthcare institutions in LMICs face considerable limitations including infrastructural constraints, significant patient load, and high patient-provider ratios, lack of orientation and training toward rational antimicrobial pharmacotherapy as well as antimicrobial stewardship amongst others (11–13). At the same time, the LMICs are also bearing the brunt of significantly high antimicrobial consumption and consequent high rates of antimicrobial resistance. Although antimicrobial stewardship programs are needed maximally in these countries, such programs are often rudimentary, where all the components necessary for successful implementation of AMS programs are seldom in place (14, 15). While in the developed world, hospital stewardship programs typically include an antimicrobial committee, continuous monitoring of anti-infective agent use and their resistance patterns and evaluation of intervention outcomes, along with development of evidence based local treatment guidelines and drug formularies. However, these components are either not present at all or exist at a bare minimum level given the above-mentioned limitations of human and organizational resources, infrastructure, and funding in LMICs.

The barriers to responsible use are several including the lack of orientation and training, diagnostic infrastructure and expertise, lack of knowledge in optimal antimicrobial prescribing and limited access to quality assured pharmaceuticals besides unique socioeconomic and cultural challenges (16, 17). These countries often lack strong political commitment that is essential to generate local evidence base for AMR as well as further development of solutions based on this data. This is critical since the factors that contribute to AMR are usually context-specific and addressing this formidable challenge requires cross-sectoral coordination engaging various government agencies, the private healthcare facilities, civil society as well as professional groups (18). In a survey carried out by Indian Council of Medical Research in 2013, it was found that among 20 tertiary level healthcare institutes, only 40% had AMS written documents, 75% had infection control guidelines, 65% had AMAs prescription guidelines, and only 30% had AMS implementation strategies (19). An international survey carried out in Africa, found that only 14% hospitals were running AMS programs (20). LMICs are considered the hotspot for both infectious diseases as well as antimicrobial resistance and yet lack of sufficient funding and expertise poses a considerable impediment for organizing functional and successful AMS programs. Other factors that may be overlooked but in fact are critical barriers in the implementation of antimicrobial stewardship activities include overcrowding, inability to follow infection prevention measures in totality, lack of electronic medical record systems, and non availability of dedicated staff. The availability of functional diagnostic laboratories is limited even in hospitals. Microbiology labs that meet the considerable requirements in terms of infrastructure, adequately trained and experienced human resource, and quality control systems in place are often few and far between especially in rural areas. When these facilities are available, the threshold for culture and susceptibility testing is usually high due to tight healthcare budgets. Other challenges include lack of culture to obtain cultures, patients often being extensively pre-treated with antimicrobials before being referred to other facilities, and a significant turnaround time for results (21, 22). This results in a vicious problem where prevalence data on the state of antimicrobial resistance is inadequate resulting in difficulties in formulating local treatment guidelines, clinicians lack essential information to guide antimicrobial selection, and finally crucial policy decisions are crippled by the lack of data highlighting the actual scale of the local, regional and national AMR problem.

Multiple studies conducted in diverse settings have highlighted inadequate knowledge and awareness regarding rational use of antibiotics among physicians and medical students in resource limited settings (23–25). Additionally in many of these countries, antibiotics are often prescribed by a wide variety of persons with diverse levels of training including healthcare workers—nurses, pharmacists, dentists, midwives, practitioners of alternative systems of medicines including herbals as well as local chemist shops, faith healers, and quacks (26–28). As outlined above, prescribers in these settings often do not have access to context-specific guidelines due to lack of familiarity with methods of guideline development as well as limited availability of appropriate data (29, 30). Even when available, the uptake of guidelines may be inadequate either due to limited dissemination or due to lack of local adaptations. Further, lack of synchronization in the recommendations of guidelines and practical issues may render the guidelines redundant.

Traditionally, LMICs have faced the problem of limited access to essential lifesaving antibiotics. However, the threat of growing AMR in these countries has been partly fuelled by the easy availability of antimicrobials as well as poor quality of available pharmaceuticals (31). As far as widespread availability of antibiotics is concerned, there is rampant misuse of nonprescribed antimicrobials that can be easily purchased over the counter as well as through the internet. Studies from various LMICs have demonstrated widespread misuse in terms of antibiotic dispensing without prescriptions which is often seen as the most convenient and affordable treatment option (20, 32). Additionally, pharmaceutical industry push through financial incentives to the prescribers especially in the poorly regulated private healthcare sector in LMICs has been ascribed to be one of the significant drivers for antibiotic over prescription (33, 34). In many LMICs, the problem of substandard and falsified medical products is pervasive and rampant. This may be a significant driver for AMR since suboptimal antimicrobial exposure is known to promote selection of resistant pathogens. In general, the confidence in the quality of available generic medicines is poor as far as healthcare workers as well as general public are concerned. A recent ministry of health survey in India, found that more than 10% drugs in the government supply chain are substandard, labeled as “not of standard quality—NSQ” (35). This widespread distrust results in prescription of more expensive, branded products and even bigger burden on the affected patients. Additionally, fixed dose combinations, which are often not rational, make unnecessary use of antimicrobials a bigger problem. Governments in most LMICs are faced with the dilemma of choosing between stringent checks on antimicrobial supply chains thus scuttling their easy availability and allowing the existing affordable system of healthcare. Inadequate public awareness regarding the threat posed by growing antimicrobial resistance may underlie patient pressure and demands for antibiotics as well as common practice of antibiotic sharing in the LMICs. Access to antibiotics without prescription through online pharmacies/internet is another potential threat which can become even bigger given the extensive penetration of mobile internet even in remote areas across the country (36).

**Figure 1:**
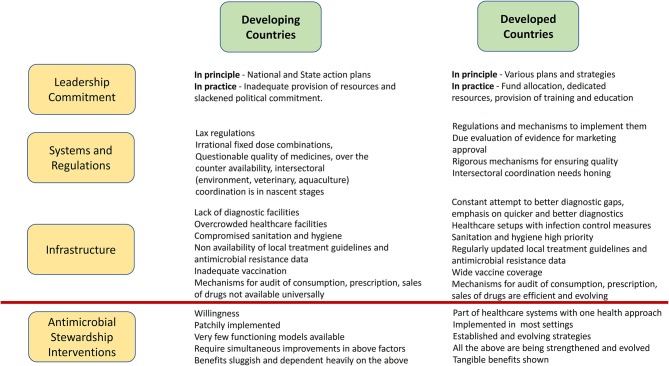
Key differences between developing and developed countries that can affect implementation of AMS programs in LMICs.

While on one hand, excess of antimicrobial use is a problem, on the other, there may be issues with access to some essential antimicrobials. Cefazolin, penicillin, amoxicillin, cloxacillin are notable in this regard. Non availability of these antimicrobials compels physicians to use higher end antimicrobials (37). Key differences between developing and developed countries which may affect successful implementation of AMS programs in LMICs are summarized in Figure 1.

Needless to say, success of AMS initiatives in these settings can be ensured only in the presence of strong political will, stringent regulatory oversight as well as concerted efforts to align policies as well as public sentiments with the objectives of antimicrobial stewardship. Over the past 6 years we have put concerted efforts into developing a functional antimicrobial stewardship program at our healthcare institution. In one of the pilot studies, a system comprising of a generic “prospective audit and feedback” form for data capture, electronic capture and analysis of records, and identification of major intervention points followed by AMS strategy implementation was applied in the surgical recovery unit within the hospital. We found significant reductions in the double anaerobic coverage, average number of antimicrobials prescribed per patient, as well as a decline in the defined daily doses (DDD) of designated antimicrobials within the study unit (38). In another study designed to assess the impact of AMS interventions on antimicrobial prescription in the tertiary care trauma center, significant improvements were noted in terms of duration, choice, indications and the route of administration of antimicrobials (39). Combined with the approach of monitoring of infection control practices, it was shown that further gains could be achieved in terms of DDD of designated antimicrobials, days of therapy (DOT) per 1,000 patient-days (PD) and length of therapy (LOT) per 1,000 PD (Data communicated for publication). Other healthcare centers and hospitals have also demonstrated the benefits of implementing antimicrobial stewardship programs in terms of improved compliance with antibiotic policies and guidelines, decline in the use of designated antimicrobials, reduction in DDD/1,000 PD as well as mean monthly costs of antimicrobial use (40, 41).

**Figure 2:**
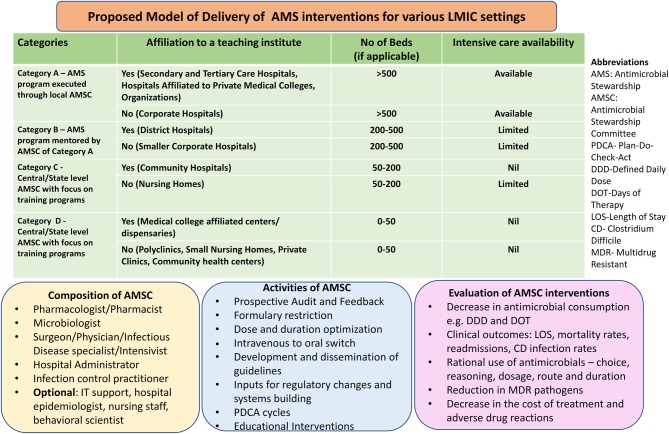
Proposed model of delivery of AMS interventions for diverse LMIC settings.

In the following sections, we outline how operationalization of various elements of antimicrobial stewardship programs may be achieved despite the barriers as understood above. We have drawn from our own experience as well as from related experiences as derived from published literature. A proposed model of delivery of AMS interventions for diverse healthcare settings in LMICs is summarized in Figure 2.

## Prospective Audit and Feedback

As per the guidelines of Infectious Disease Society of America, prospective audit and feedback is one of the two core strategies recommended for successful antimicrobial stewardship programs (8). A typical prospective audit and feedback activity requires a cross functional team comprising of a physician usually an infectious diseases specialist, a microbiologist and clinical pharmacists (7). Several studies have shown that these roles are not sacrosanct and various healthcare professionals with adequate expertise and motivation can be trained optimally to perform AMS activities. Seto et al. demonstrated that a trained nurse could take on the role of the pharmacist, whereas another group successfully employed pharmacy residents and students for the same role (42–44). Across the globe, pharmacists constitute the third largest health care professional group, however given the barriers faced in LMICs, the profession still has a long way to go in order to strengthen and contribute substantially in the development of health care systems (45). A systematic review has shown that current evidence-base highlighting the quality of professional services delivered by the pharmacies in LMICs is limited. The available literature indicates that standards are often suboptimal including the lack of presence of adequately trained pharmacists, the provision of advice for common ailments not being in accordance with evidence based guidelines and often inappropriate dispensing of pharmaceuticals (46). The challenges faced in our settings are similar, therefore we have utilized the services of clinical pharmacologists for providing dedicated prospective audit and feedback services in our healthcare settings. A clinical pharmacologist or pharmacologist is uniquely positioned to undertake activities performed both by a pharmacist as well as physician since they, by training and education, are skilled to understand the nuances of antimicrobial pharmacotherapy as well as are well aware of antimicrobial PK/PD, drug-drug and drug disease interactions. Their training as medical graduates helps in assessment of possible etiologies, organisms, susceptibility patterns, severity of infections, need for source control, as well as reasonable diagnostics required. Given the grossly inadequate doctor-patient ratio in the developing world and often the lack of interest and/or availability of ID physicians, role of a clinical pharmacologist is poised to expand if AMS endeavors are to be successful in resource constrained environments. Similarly, clinical microbiologists who are by training medical doctors can be leveraged to provide these services. An approach of team based delivery of appropriate inputs for managing infections can be developed, particularly in case of hospitals affiliated to training centers. The team can be expanded to include medical graduates in community medicine and public health disciplines to extend the services to health centers which are directly or indirectly affiliated and are largely offering outpatient services or minimal inpatient services.

Generally, in a one-step prospective audit and feedback, a AMS team member accompanies and provides direct feedback during clinical rounds often focusing on irrational use of high-end antibiotics. In the more intensive two step method, an AMS team member will review the cases from a particular ward or intensive care unit of the healthcare facility individually. Subsequently, the cases meeting the criteria for intervention will be presented to a senior team member who after vetting, will communicate the AMS team recommendations for treatment modification or discontinuation of antibiotics to the treating physician through written or direct verbal advice (42, 47, 48).

The principle advantage of a prospective audit and feedback practice is that prescribers do not perceive the loss of autonomy and it is consequently far more acceptable to the doctors. Additionally in resource constrained settings, the strategy also provides opportunities for regular physician education and real time discussions, and importantly it may be customized to the size of the facility depending on the resources available. Often the initial target areas are intensive care units and other wards with high antimicrobial consumption. Once antibiotic prescriptions in these areas are deemed to be streamlined as documented through consumption patterns, the focus of AMS activities may be shifted to newer or other problematic areas. Another advantage is individualization of therapy allowing drug interactions, socioeconomic considerations and individuals' clinical conditions to be taken into account.

At our organization, we undertook the prospective audit and feedback as one of the earliest AMS interventions (49). At the outset, a paper form was devised for capturing the relevant patient, prescription, laboratory, culture and sensitivity data in adequate details. This was followed by multiple pilot runs and discussions with all the stakeholders including physicians, surgeons, microbiologists, and pharmacologists, which led to several iterations of the original form to ultimately reach its current shape. While worldwide, particularly in the developed country settings, source of such data is usually the hospital pharmacy. However, there were several roadblocks to achieve this purpose in our case, principle being the fact that the hospital pharmacy in developing countries caters partially and to the need of only a minority of the patients with several drugs being bought individually through out of pocket expenditures. Further, this would provide only a quantitative estimate of the antimicrobial consumption and the quality/appropriateness of the antibiotic prescriptions could not have been assessed. Taking these considerations into account, we designed a form that had multiple sections for tracking the antimicrobials prescribed, their rationality with respect to choice of agent, dose, and duration of the therapy, the microbiological data, and patient outcomes—both clinical as well as microbiological. Concurrently, we developed an online electronic system for transcribing and evaluating the antimicrobial prescription data. In the initial phase, only residents from the pharmacology department were assigned the job of completing the record forms and presenting the reports to AMS team members. However, given the need to ensure regular inflow of data and periodic feedbacks, residents in three selected units were asked to fill out the AMS audit forms for the admitted patients on a daily basis. These forms were collected by the pharmacology residents for collating, evaluating, and informing the findings to the individual units. The key intervention points guiding the strategic decisions for each participating unit were identified followed by the AMS interventions and evaluation of outcomes. The key interventions were assisting residents with selection of appropriate antimicrobial, and its dose and duration of therapy. The nature of these interventions was suggestive with the final decision on their uptake lying with the consultant in charge of the unit. Suggestions were also made regarding the optimal doses, discontinuation of redundant antibiotics, escalation or addition of an antibiotic on a case-by-case basis. The PAF interventions led to a significant decline in double anaerobic coverage, the average number of antimicrobials prescribed per patients and a decrease in DDD of designated antimicrobials. Additionally, the utilization of optimized doses increased (38). This pilot system functioned with watchful oversight by unit in charge. This was done purposely since it was deemed that participatory and contributory decision making is critical to the long term sustenance and success of the program with younger prescribers more likely to accept and emulate the interventions well received by their more experienced seniors.

The common limitations of the PAF include it being a labor-intensive strategy required trained manpower with familiarity to the principles of antibiotic prescribing as well as the clinical conditions affecting antimicrobial choices, doses as well as durations (50, 51). It has been realized that routine curricula and learning in medicine, pharmacology, pharmacy as well as nursing disciplines does not emphasize adequately on the principles of antibiotic prescribing. Thus, specific training in the discipline of AMS is essential before qualified individuals can undertake these activities (52). Another disadvantage is that the physician acceptance of recommendations made by the AMS team is often voluntary, thus limiting the overall effectiveness of PAF as compared to front end strategies such as formulary restriction and preauthorization. Moreover, the prescribers may not be very receptive to the recommendations of the AMS team especially if the patient is doing well on their prescribed antimicrobials, however broad spectrum they may be. Some may even question the dependability of the advice especially if the patients are not actually seen by the AMS team members. All these well described limitations of PAF are often magnified in the healthcare settings prevalent in the lower and middle income countries as described above. Thus, continuous, coherent and persistent efforts are required as part of a multipronged strategy to curb antimicrobial resistance successfully.

## Education

One of the most basic and effective tool to influence prescriber behavior is education sessions that inform as well as engage clinicians and other health care professionals in stewardship activities. These educational sessions may take a formal shape as continuing medical education presentations, group teaching sessions, and mobile/email alerts and notices. Informal modes of education on the other hand include, impromptu bedside teaching by stewardship team members or during PAF rounds (53). As far as content of these sessions is concerned, they can either focus on general AMS principles, rational antibiotic prescribing for specific indications or clinical conditions/comorbidities, and supportive activities such identifying drug allergies; inculcating culture of cultures; interpretation of culture and susceptibility reports; indications for use of high end/ designated antimicrobials; understanding and applications of antibiograms; use of local/ institutional guidelines, appropriate filling of forms and/or documentation, and feedback of audit results. If a new AMS intervention is being rolled out, its success is often contingent upon adequate and timely education sessions focussing on the target group of healthcare professionals. These educational sessions can also be used to present specific data on antimicrobial use in those wards/intensive care units and to point out improvements or worsening of prescription trends. Such activities have been shown to motivate and support improved antimicrobial prescribing practices. It must be noted, however, that when used in isolation, educational sessions have negligible influence and they must be accompanied by corresponding interventions and measurement of outcomes to have a significant impact on prescribing behaviors. In addition, reiteration of educational messages and sessions is necessary to sustain any gains in antimicrobial prescribing practices especially in settings where there is a high turnover of healthcare personnel (8, 54).

We have been carrying out several educational activities under the umbrella of our AMS including but not limited to case discussions—routine as well as special, AMS rounds with bite sized information sessions given bedside, focussed didactic sessions, disseminating antimicrobial snippets through staff/work mobile phones as well as dedicated nurse demonstrations. In addition, day long continuing medical education and training programs for healthcare professionals as well as general public and extending support to other healthcare facilities keen on initiating and/or functionalizing their AMS programs are also being undertaken (49).

Inappropriate antimicrobial dosing includes a range of problems including overdosing, under dosing, failure to adjust doses in special clinical situations or to employ loading doses wherever required, or issues with frequency of dosing. Errors in antimicrobial dosing are often rampant in LMICs as highlighted above primarily due to inadequate emphasis on optimal antibiotic prescribing in undergraduate and postgraduate medical curricula. These errors besides promoting antimicrobial resistance can also increase the possibility of patient harm due to adverse effects or therapeutic failure. To address these issues, information and education regarding selection of optimal antibiotic doses is provided on as need basis. The process of establishing the optimal doses for patients in a particular setting for selected antimicrobials has been started. Besides, training for rational and judicious use of antimicrobials is provided through weekly case discussions during multidisciplinary rounds involving clinicians, microbiologists as well as clinical pharmacologists. The clinical team is encouraged to send queries related to specific admitted inpatients details of which are then presented and discussed in these rounds. The AMS team strives to provide up to date, evidence based and unbiased information supporting selection of treatment regimen to guide further clinical management of the patient in question. These exercises have been instrumental in minimizing inappropriate use of antimicrobials, tailoring the antibiotic therapy to individuals needs wherever required and improving patient outcomes while simultaneously educating and training the prescribers. Although educational sessions are often manpower and labor intensive, they have been shown to improve prescribing behavior as well as improve acceptance and uptake of other AMS strategies in the healthcare facility (53, 54).

## Optimization of Surgical Antibiotic Prophylaxis

Optimal administration of perioperative antimicrobials is a high yield intervention and often considered a low hanging fruit and a good starting point for initiating AMS activities in institutions. Studies have demonstrated improvements in patient outcomes in terms of reduced incidence of surgical site infections, lower morbidity and mortality, costs as well as lower rates of *C. difficile* infections (55). The optimization process entails two major objectives—selection of appropriate antibiotic and dose that is administered timely before and during surgery and limiting unnecessary and prolonged prescription of antimicrobials in the postoperative period (56). Implementation of this strategy requires facility wide audit to document adherence to guidelines developed by various major societies/agencies. This is usually followed by actions to improve practices in areas where they are nonadherent to prescribed guidelines. Once the practices have been optimized, periodic monitoring is necessary and feedback is necessary to sustain benefits derived from improved preoperative prophylaxis.

In our institute, we carried out an initial audit aimed to understand the pattern of surgical prophylaxis practices across the healthcare facility. We observed wide variations in prescription practices not only among different departments but also within the same department among various units. Further, the antimicrobials were being continued for durations much longer than recommended and the choice of antibiotics was also not in consonance with the society guidelines. The clinicians made these choices based on their apprehension of inadequate sterilization techniques, environmental contaminants, and lack of availability of antimicrobial sensitivity data to support the recommended practices. The susceptibility data, although, being circulated periodically remained underutilized in policy formulation. With this background, the AMS team decided to start the stewardship within a single surgery unit, where it was proposed that suggested modifications of the prophylaxis regimen will be followed. The patients were followed for the development of surgical site infections as well as hospital acquired infections. Though surgical prophylaxis is not directed against hospital acquired infections, we included it as a part of confidence building measure. The data so obtained was discussed with the faculty members of the unit. It was then suggested that they develop a consensus guideline with all the consultants participating in the process. Following the development and implementation of the guidelines, a system for conducting regular audits was simultaneously initiated. These audits not only help in assessing the extent of adherence but also analyzing and subsequently addressing the reasons behind any future deviations (49, 56).

## Development of Evidence-Based Guidelines

One of the significant activities under successful AMS programs is the development of multidisciplinary evidence based antibiotic guidelines based on locally generated data (8). Such evidence based guidelines lead to standardized and quality patient care by facilitating selection of initial therapy for wide variety of commonly encountered infections in clinics and wards. These guidelines are devised to suggest both first as well as alternative antimicrobials for common infections in the ICU, ward as well as outpatient departments. The selection of agent is usually based in the site of infection, the usual pathogens suspected, the local data on epidemiology and resistance patterns, evidence on sensitivity and effectiveness, AMS principles, and agent availability in the hospitals (57). Needless to say, empirical antibiotic guidelines are appropriate in most clinical situations but prescribers are always advised to consider patient specific information while considering the suitable antimicrobial therapy for their patients. One of the efficient strategies to develop empiric antimicrobial therapy guidelines at the institute level is to employ established society and/or national guidelines and to adapt it suitably to meet the local requirements and resistance patterns. However, they need to be revised regularly as and when new data becomes available for consideration. The acceptability and uptake of guidelines is significantly facilitated by the involvement of local stakeholders and also provides a sense of ownership to the prescribers. Regular dissemination of guidelines among all the prescribers is necessary to ensure compliance with these documents.

A typical intensive care unit (ICU) environment is considered ideal for selection and spread of multidrug resistant organisms on account of several factors viz. critically ill patients, numerous opportunities for cross infection and intensive use of antimicrobials. While prompt, targeted and accurate selection of antibiotics is ideally warranted, the initial choice is often empiric, use of broad spectrum antimicrobials which if not rationally chosen can potentially select multidrug resistant bacteria. This often sets up a vicious cycle of antibiotic use where each new prescription adds to selection pressure for even more resistant phenotypes. Thus, appropriate selection of antimicrobial therapy for ICU patients is pivotal not only to ensure that critically ill and immunosuppressed patients receive effective and safe pharmacotherapy but also to contain resistance selection that can have beneficial spillover effects for the entire healthcare facility.

In our institute, it was felt that since ICUs are major areas where high end and often multiple antimicrobials are used for prolonged durations in critically ill patients, it is crucial to have a guidance document to enable the residents and consultants make their antibiotic use rational. Since the profiles of patients presenting to individual ICUs are not homogenous, it was thought that the best way to go about it would be to have ICUs internally discuss and formulate a policy suited best for themselves. They were encouraged to seek advice from AMSC during the process. In follow up of this, the ICU representatives made a presentation of the policies developed by internal consensus. The comments and suggestions made during the meeting were communicated to the ICU in-charge and the nodal persons. These were appropriately addressed and a revised policy was submitted for finalization. In this meeting, the representatives of various ICUs put forth the idea that mechanisms should be set in place for wide dissemination of these guidelines and audit and feedback procedures. It was clarified that the guidelines were not intended to replace the critical evaluation and judgement that underpin the formulation of appropriate treatment plan in an individual case. Users of antibiotic policy document were advised to critically assess the content of guidelines in relation to local circumstances and keep themselves apprised of any changes in literature that may have occurred since the release of the document. Additionally, individual intensive care units were advised to revise these guidelines at a period of not more than 2 years on the basis of their own antibiograms.

## Conclusions and Future Directions

There are several existing roadblocks plaguing antimicrobial stewardship programs in resource constrained environments and the current evidence base supporting various AMS strategies in LMICs is scanty. However, proven AMS interventions need to be contextualized before they can be successfully employed in LMICs and they must undergo rigorous testing to support their application on a wider scale. Given the complexity and challenges of implementing various AMS program strategies in resource limited settings, it is advisable to rely on plan-do-check-act (PDCA) model to improve the efficiency as well as the likelihood of success of the program. As in other situations where applied, this iterative PDCA cycle results in quicker troubleshooting and continuous improvements in the system. Besides the activities and challenges highlighted above, other critical issues such as development of electronic health record systems and clinical decision support systems, development of adequately skilled and trained human resource, enabling environment for infection prevention strategies, addressing the conundrum of access vs. excess and political commitment with sustained and enhanced financing, need to be prioritized if the overarching goal of global containment of antimicrobial resistance is to be achieved.

## Author Contributions

AK and NS conceived the original idea and wrote the manuscript with inputs for other members of the author team. GS, NS, PR, VG, RA, JM, and PA contributed to the design and implementation of the AMS strategies in our institute. GS, PR, and JM helped supervise the project.

## Conflict of Interest

The authors declare that the research was conducted in the absence of any commercial or financial relationships that could be construed as a potential conflict of interest.
